# A weaker donor shows higher oxidation state upon aggregation†

**DOI:** 10.1039/c8ra02956c

**Published:** 2018-05-11

**Authors:** Longfei Ma, Haili Peng, Xiaofeng Lu, Lei Liu, Xiangfeng Shao

**Affiliations:** State Key Laboratory of Applied Organic Chemistry, Lanzhou University Tianshui Southern Road 222 Lanzhou Gansu Province P. R. China shaoxf@lzu.edu.cn +86 0931 8915557 +86 0931 8912500

## Abstract

The charge-transfer between TTFs and I_2_ shows that the stronger donor TTF1 is in a cation radical state and the weaker donor TTF2 is neutral in solution, whereas TTF1 exists as a cation radical and TTF2 is dicationic in complexes. The dicationic and neutral states of TTF2 are reversible upon aggregation and solvation.

## Abstract 2

A weaker donor is dicationic but a stronger donor appears as a cation radical in their CT complexes with iodine.

## Graphical abstract

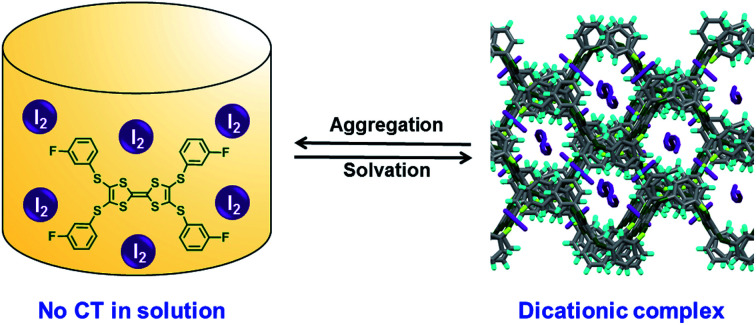

Charge-transfer (CT) between an electron donor and acceptor plays the pivotal role in supramolecular assembly and creation of conducting materials. There remains a challenge in CT, that is, whether a weaker donor could show a positively charged state higher than a stronger donor through the CT with the same acceptor.

Iodine (I_2_) can serve as an acceptor to prepare CT complexes. The CT complex perylene–iodine is one of the earliest organic conductors.^1^ Upon gaining one electron from a donor molecule, iodine would form polyiodides,^2^ which show diverse structures and have received growing interest in supramolecular architectures and materials science.^3,4^ Tetrathiafulvalene (TTF) is an electron donor with three reversible states, (TTF)^0^, (TTF)^+^˙, and (TTF)^2+^.^5^ TTF derivatives (TTFs) have been widely employed as building blocks for functional materials.^6^ The CT complexes of I_2_ and TTFs can be prepared by mixing these two species.^7^ Because I_2_ is not a strong acceptor, TTFs are mainly in the cation radical or partially charged state in CT complexes.^8^ Ar-S-TTFs are derived from TTF by decorating four arylthio groups onto the peripheral positions (Scheme 1). Ar-S-TTFs can adjust their geometry and electronic state to adapt to a guest molecule,^9^ and they form CT complexes with various acceptors such as fullerene,^10^ heteropoly acid,^11^ and CuBr_2_.^12^

**Scheme 1:**
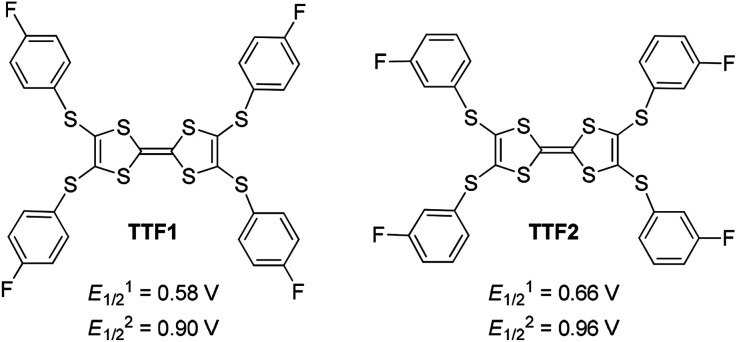
Chemical structures of the Ar-S-TTFs reported herein, along with their first (*E*_1/2_^1^) and second (*E*_1/2_^2^) redox potentials in CH_2_Cl_2_ recorded *versus* SCE.

The structures of polyiodides depend on the nature of the counter cations,^3*b*^ and Ar-S-TTFs can modulate the geometry and electronic state according to the guest. Therefore, the CT complex containing these two flexible components seems promising. Being continuous study on Ar-S-TTFs, herein we report the CT between Ar-S-TTFs (TTF1 and TTF2) and I_2_. It is found that a weaker donor TTF2 carries the positive charge higher than a stronger donor TTF1 in their CT complexes with I_2_. Meanwhile, the iodine atoms form polyiodides with different structures in CT complexes, *i.e.*, the infinite covalent chain of [(I_*n*_)^−^]_∞_ in TTF2 complex and 2-D network comprised of (I_3_)^−^ and I_2_ in TTF1 complex.

Electrochemical analysis shows that both TTF1 and TTF2 have two reversible redox potentials. The first redox potential (*E*_1/2_^1^) of TTF2 (0.66 V *vs.* SCE in CH_2_Cl_2_) is higher than that of TTF1 (0.58 V), and the second redox potentials (*E*_1/2_^2^) show similar tendency (Scheme 1). Therefore, as donor molecule, TTF2 is weaker than TTF1. Both donors display weak absorption band at 400–500 nm due to the intramolecular CT transition,^9^ whereas the cation radicals of them show broad absorption at 650–1100 nm.^11^ For example, electrochemical oxidation of TTF1 under constant potential of 0.75 V results in an absorption band in this region as proved by the spectroelectrochemical study (Fig. 1a).

**Fig. 1:**
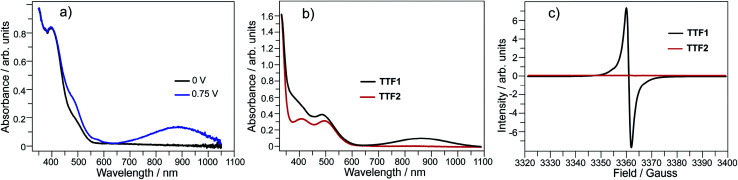
(a) Spectroelectrochemistry of TTF1 in CH_2_Cl_2_ (*c* = 5 × 10^−4^ mol L^−1^); (b) UV-Vis absorption spectra and (c) ESR spectra of TTF1 and TTF2 upon adding 3 equivalents of I_2_ in CH_2_Cl_2_ (*c* = 1 × 10^−5^ mol L^−1^).

By mixing TTF1 and I_2_ in CH_2_Cl_2_, an absorption band appears at 650–1100 nm (Fig. 1b), which is identical to that observed in the spectroelectrochemistry. The mixture of TTF1 and I_2_ in CH_2_Cl_2_ shows ESR signal with *g* = 2.006 (Fig. 1c). Therefore, the CT occurs between TTF1 and I_2_ in CH_2_Cl_2_ solution, and TTF1 is at the cation radical state. While CT occurs between TTF1 and I_2_ in CH_2_Cl_2_, the thin layer chromatography reveals that the neutral TTF1 remains in solution even though excess I_2_ is added (>3 equiv.); this means I_2_ cannot completely transform TTF1 into cation radical. On the other hand, there is no CT between TTF2 and I_2_ in CH_2_Cl_2_ solution, because the absorbance of (TTF2)^+^˙ is not observed (Fig. 1b) and the mixture of TTF2 and I_2_ is ESR inactive (Fig. 1c).

Although TTF1 and TTF2 exhibit the different behaviors upon mixing with I_2_ in CH_2_Cl_2_, they both afford CT complexes with I_2_. The CT complexes are obtained as black block-like single crystals by evaporating the CH_2_Cl_2_/*n*-hexane (v/v, 1 : 1) solution of mixture of TTF1 (or TTF2) and I_2_ at room temperature. The compositions of complexes are determined on the basis of single crystal structure analyses to be (TTF1)·(I_3_)·(I_2_) and (TTF2)·(I_5_)·(I_2_).

(TTF1)·(I_3_)·(I_2_) crystallizes in the *P*1̄ space group. There are one TTF1 molecule and three pairs of iodine atoms (I1–I2, I3–I4, and I5–I6) in the asymmetric unit. The I3 and I5 locate on the inversion centres. The bond length of central C=C (bond *a* in Scheme 2) on TTF moiety can be used to estimate the charge on TTFs,^7^*i.e.*, 1.34 Å, 1.39 Å, and 1.45 Å respectively for (TTF)^0^, (TTF)^+^˙, and (TTF)^2+^. Referring Fig. 2a, the central C=C bond length in TTF1 is 1.39 Å, same to that in (TTF)^+^˙.^7^ The site charge (*ρ*) on TTF moiety also can be estimated *via* an empirical formula *ρ* = 6.347 − 7.436*δ*,^13^ where *δ* = (*b* + *c*) − (*a* + *d*), and *a*, *b*, *c*, and *d* are bond lengths (Scheme 2). The calculated *δ*-value of TTF1 is 0.721 Å, which gives the site charge on TTF1 to be +1. The iodine atoms (I1–I6) form three tightly connected units, [I1–I2], [I4–I3–I4], and [I6–I5–I6] (Fig. 2c). The I1–I2 bond length (2.74 Å) is identical to that of neutral I_2_ (2.74 Å), and the I–I bond lengths (2.91–2.93 Å) in both [I4–I3–I4] and [I6–I5–I6] are very close to that of triiodide (2.90 Å).^14^ Therefore, the [I4–I3–I4] and [I6–I5–I6] units are intrinsic (I_3_)^−^. These results indicate that TTF1 is at cation radical state in complex, which is reasonable according to the formation of (TTF1)^+^˙ in solution by mixing TTF1 and I_2_.

**Scheme 2:**
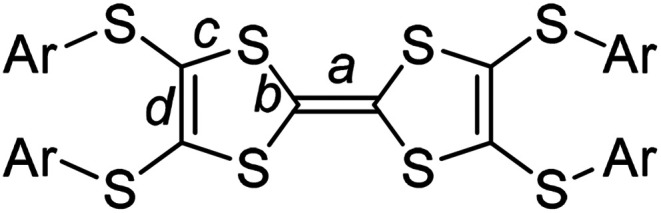
The bonds (*a*–*d*) on Ar-S-TTFs for the estimation of charge *ρ*.

**Fig. 2:**
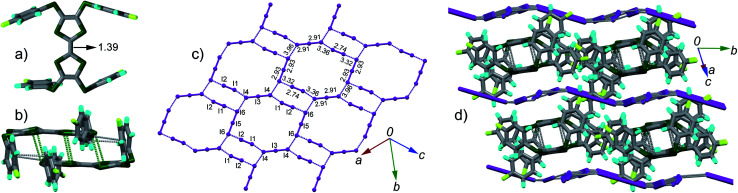
Crystal structure of complex (TTF1)·(I_3_)·(I_2_): (a) top view of molecule TTF1 with the central C=C bond length shown in unit of Å; (b) TTF1 dimer with atomic short contacts shown in dashed lines (green for S⋯S and grey for C⋯S); (c) anion sheets composed of (I_3_)^−^ and I_2_ with the I–I bond length and I⋯I contacts (purple dashed lines) shown; (d) packing structure viewed along the longitudinal axis of TTF1 dimer with the I⋯I contacts shown in grey dashed lines.

The TTF1 molecules are dimerized in complex (Fig. 2b). Within a dimer, there are S⋯S contacts (3.45–3.53 Å) between TTF cores, and C⋯S contacts (3.42–3.46 Å) between the peripheral sulfur atoms and the phenyls. Meanwhile, the (I_3_)^−^ anions and neutral I_2_ together form the two-dimensional (2-D) sheet *via* multiple I⋯I contacts (3.32–3.96 Å). The 2-D sheet is not flat but shows a zig–zag shape along the *b*-axis direction (Fig. 2d). The dimers of TTF1 are sandwiched by the neighbouring 2-D anion sheets. There are I⋯S contacts (3.69–3.78 Å) between the anion sheets and TTF1 dimers. This type of 2-D polyiodide framework is rare in the CT complexes of TTFs and I_2_.^15^

(TTF2)·(I_5_)·(I_2_) crystallizes in the *C*2/*c* space group. The asymmetric unit contains half of TTF2, three tightly connected iodine atoms (I1, I2, I3) with I3 on the 2-fold screw axis, and one isolated iodine atom (I4) at the general position. Referring Fig. 3a, the central C=C bond length (1.45 Å) on TTF moiety is close to that observed in the dicationic salts of Ar-S-TTFs (1.42 Å).^12^ The calculated *δ* value of TTF2 is 0.573 Å, giving the site charge on TTF2 to be +2. These results firmly prove that TTF2 is dicationic in complex, against the neutral state of TTF2 by mixing it with I_2_ in CH_2_Cl_2_. As shown in Fig. 3b, the I4–I4 bond length (2.79 Å) is close to that of I_2_ (2.73 Å), thus the (I4)_2_ is a neutral I_2_. The I1, I2, and I3 atoms form an infinite chain with a periodicity of –[I1–I2–I3–I2–I1]–. Regarding the charge on TTF2, a periodic unit [I1–I2–I3–I2–I1] has a charge of −2. The interatomic distances in [I1–I2–I3–I2–I1] unit vary from 3.04 Å to 3.19 Å, almost identical to those in the infinite polymeric [(I_*n*_)^−^]_∞_ (3.02–3.20 Å).^3*a*^ Therefore, the present polyiodide chain also would be a [(I_*n*_)^−^]_∞_ polymer, and all the iodine atoms in [(I_*n*_)^−^]_∞_ are partially charged.^3*a*^ The [(I_*n*_)^−^]_∞_ chains are connected by (I4)_2_ through the I⋯I contacts (3.42 Å) to form a ladder-like structure. The TTF cores and peripheral aryls on TTF2 molecules together form a channel along the longitudinal axis of TTF2 (Fig. 3c), and the channel grows through the C⋯S contacts (3.34–3.48 Å) between the peripheral sulfur atoms and the phenyls. The [(I_*n*_)^−^]_∞_ chains penetrate into the channel. It is worth noting that [(I_*n*_)^−^]_∞_ chain has not been observed in the complexes comprised of TTFs and polyiodide.

**Fig. 3:**
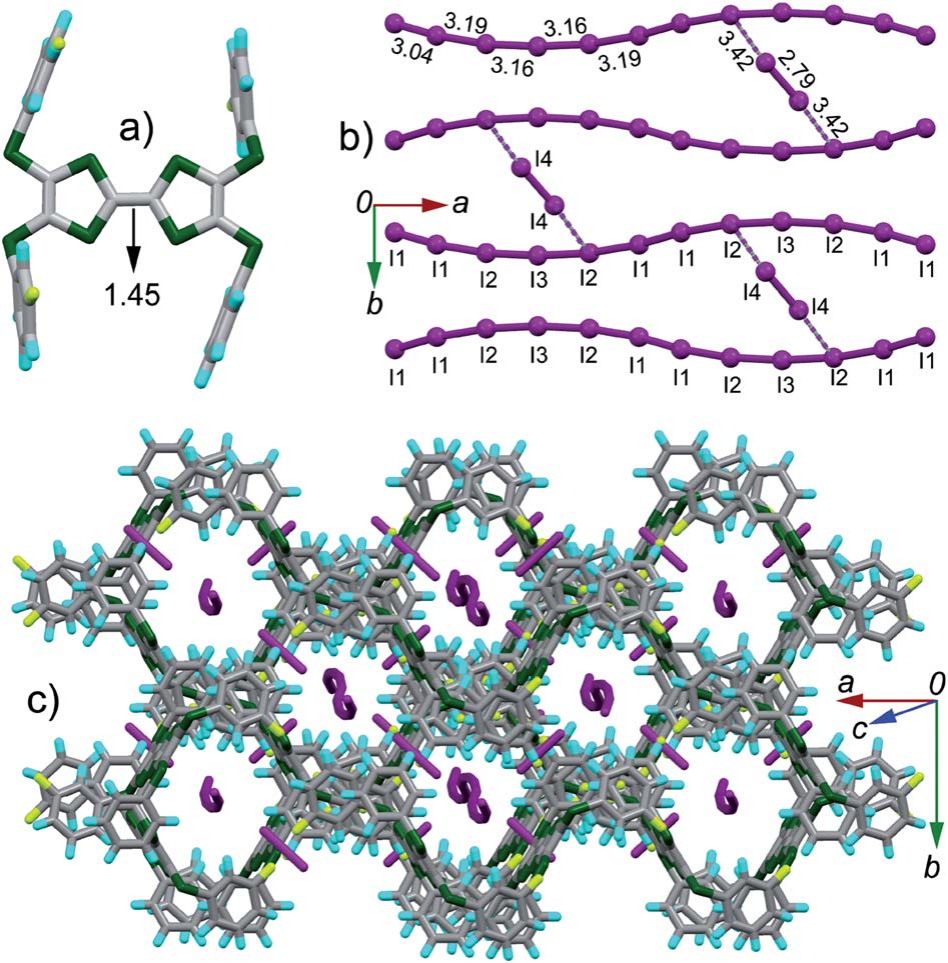
Crystal structures of complex (TTF2)·(I_5_)·(I_2_): (a) top view of molecule TTF2 with the central C=C bond length shown in unit of Å; (b) the (I_3_)^−^ anion chain with the I–I bond lengths and I⋯I contacts (purple dashed lines) shown; (c) packing structure projected along the longitudinal axis of the TTF moiety.

The charged states of TTF1/TTF2 in CT complexes are further proved by the spectroscopic studies. (TTF1)·(I_3_)·(I_2_) shows a ESR signal with *g* = 2.009 and (TTF2)·(I_5_)·(I_2_) is ESR inactive (Fig. 4a). This is consistent with crystallographic study, *i.e.*, TTF1 and TTF2 are respectively at cation radical and dicationic states. The UV-Vis absorption spectra of both complexes in solid state are distinct from those of neutral TTF1 and TTF2 (Fig. 4b). (TTF1)·(I_3_)·(I_2_) shows two absorption bands at the low energy region. The band at 800–950 nm that belonging to absorbance of (TTF1)^+^˙. The band at 950–1400 nm ascribable to intermolecular CT transition between the TTF1 cation radicals in a dimer, *i.e.*, (TTF1)^+^˙ + (TTF1)^+^˙ → (TTF1)^2+^ + (TTF1)^0^.^8*a*^ The (TTF2)·(I_5_)·(I_2_) displays very broad absorption at 500–1400 nm, which is distinct from (TTF1)·(I_3_)·(I_2_).

**Fig. 4:**
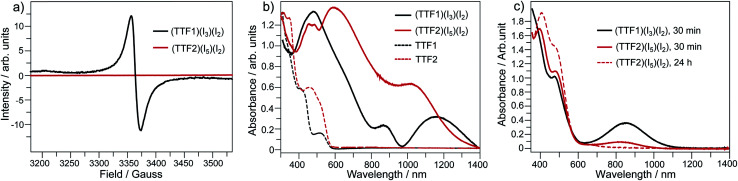
(a) ESR spectra for the crystalline complexes of (TTF1)·(I_3_)·(I_2_) and (TTF2)·(I_5_)·(I_2_); UV-Vis absorption spectra of (TTF1)·(I_3_)·(I_2_) and (TTF2)·(I_5_)·(I_2_) in the (b) solid state, and (c) CH_2_Cl_2_ solution (*c* = 10^−5^ mol L^−1^) after standing under inert atmosphere for 30 min and/or 24 h.

As aforementioned, TTF2 is neutral upon mixing with I_2_ in CH_2_Cl_2_, whereas it is dicationic in (TTF2)·(I_5_)·(I_2_). Moreover, TTF2 is a donor weaker than TTF1, but it shows higher oxidation state in complex. This is against to the criteria for CT between TTF and acceptor, say, the charge on TTF in CT complex depends on the oxidation potential (*E*^ox^_D_) of TTF and the reduction potential (*E*^red^_A_) of acceptor.^16^ The TTF would be neutral, cation radical, and partially charged under the condition of *E*^ox^_D_ − *E*^red^_A_ > 0.34 V, *E*^ox^_D_ − *E*^red^_A_ < −0.02 V, and −0.02 V < *E*^ox^_D_ − *E*^red^_A_ < 0.34 V, respectively. In the present case, the *E*^red^_A_ of TTF2 is 0.69 V and the *E*^red^_A_ of I_2_ is 0.53 V (Fig. S4 in ESI†). Therefore, TTF2 would be partially charged in CT complex. One may concern that the increment of charge transfer degree between I_2_ and TTF2 in (TTF2)·(I_5_)·(I_2_) would be attributed to the aggregation of donor and acceptor.

In this regard, the absorption spectra of complexes are studied by dissolving them in CH_2_Cl_2_. (TTF1)·(I_3_)·(I_2_) shows characteristic absorbance of (TTF1)^+^˙ in CH_2_Cl_2_ (Fig. 4c), therefore the charged state of TTF1 remain the same in solution and CT complex. On the other hand, the charge on TTF2 is distinctly variated by dissolving (TTF2)·(I_5_)·(I_2_) in CH_2_Cl_2_. The TTF2 is reduced from (TTF2)^2+^ to (TTF2)^+^˙ in 30 min as proved by an absorption band at 700–1050 nm. And, the (TTF2)^+^˙ disappears to give neutral TTF2 when the solution is kept for 24 h under inert atmosphere. This means that the retro CT occurs from [(I_*n*_)^−^]_∞_ to (TTF2)^2+^ upon dissociation of (TTF2)·(I_5_)·(I_2_), and both anionic and cationic components return to the neutral state. Moreover, the absorbance of (TTF2)·(I_5_)·(I_2_) can be restored by evaporating the solution to gain solid complex. This process, exchanging the dicationic and neutral states of TTF2, is thus reversible upon aggregation and solvation of complex as shown in Scheme 3. These results prove that the dicationic state of TTF2 in CT complex comes from the aggregation of donor and acceptor.

**Scheme 3:**
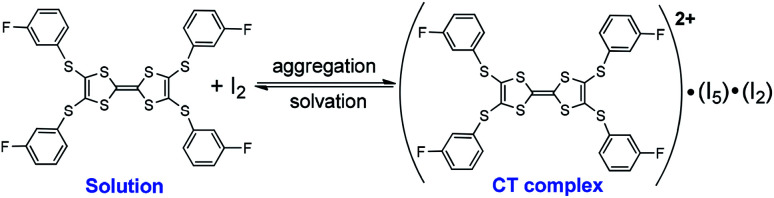
Reversible process upon aggregation and solvation of (TTF2)·(I_5_)·(I_2_)

In summary, the CT between TTF1/TTF2 and I_2_ is studied in both solution and solid state. The stronger donor TTF1 turns into cation radical and the weaker donor TTF2 remains neutral upon mixing with I_2_ in solution. On the other hand, TTF2 shows an oxidation state (dicationic) higher than that of TTF1 (cation radical) in their CT complexes, which is unusual for CT between TTFs and acceptors. The high oxidation state of TTF2 in complex is due to the aggregation of donor and acceptor. The dicationic and neutral states of TTF2 are reversible upon aggregation and solvation of CT complex. Moreover, the structures of polyiodides in CT complexes can be finely tuned by varying the aryls on Ar-S-TTFs, to give infinite [(I_*n*_)^−^]_∞_ and 2-D network comprised of (I_3_)^−^ and I_2_. Along with previous report, this work further indicates that Ar-S-TTFs show unique feature, *i.e.*, self-modulation of electronic states and molecular geometries according to guest molecules.

## Conflicts of interest

There are no conflicts to declare.

## Supplementary Material

RA-008-C8RA02956C-s001

RA-008-C8RA02956C-s002
