# Supplementary material for: A weaker donor shows higher oxidation state upon aggregation
Source: RSC Adv. 2018 May 11;8(31):17321–4. doi: 10.1039/c8ra02956c (PMC9080441; doi:10.1039/c8ra02956c)
Supplement: RA-008-C8RA02956C-s001 [file RA-008-C8RA02956C-s001.pdf]

Electronic Supplementary Material (ESI) for RSC Advances.
This journal is © The Royal Society of Chemistry 2018

# Supporting Information

## General methods

The **TTF1** and **TTF2** were synthesized according to our previous report, and they all were triply recrystallized from appropriate solvents to gain high purity before conducting the experiments herein. The solvents were purified by following the standard procedure.

UV-Vis spectra of Ar-S-TTFs in $CH_2Cl_2$ solution ($10^{-5}$ mol $L^{-1}$) were measured on UV-2006 UV-Specterophotometer at 20 °C. The solid state UV-Vis spectra were measured by dispersing the samples on the KBr pellet. Cyclic voltammetry of Ar-S-TTFs was recorded on a RST 5000 electrochemical workstation at a scan rate of 50 mV $s^{-1}$, with glassy carbon discs as working electrode, Pt wire as counter electrode, and SCE electrode as reference electrode. The concentration was $5\times10^{-4}$ mol $L^{-1}$ in $CH_2Cl_2$ and the supporting electrolyte was $(n\text{-Bu})_4N\bullet PF_6$ (0.1 mol $L^{-1}$). The measurement was performed at 20 °C after bubbling the solution with $N_2$ gas for 15 min. The *in-situ* investigation of the absorption spectra of Ar-S-TTFs under constant potential electrochemical-oxidation was performed on a Zahner CIMPS type photo-electrochemical workstation. ESR spectra were measured on a Bruker ER200DSRC10/12 device with microwave frequency of 9.444 GHz and power of 24.127 mW. The single-crystal X-ray diffraction was performed at 173 K on a SuperNova (Agilent) type diffractometer. The crystal structure was solved by direct method *SIR*2004 and refined by full-matrix least-square method on $F^2$ by means of *SHELX*L-97. The calculated positions of the hydrogen atoms were included in the final refinement.

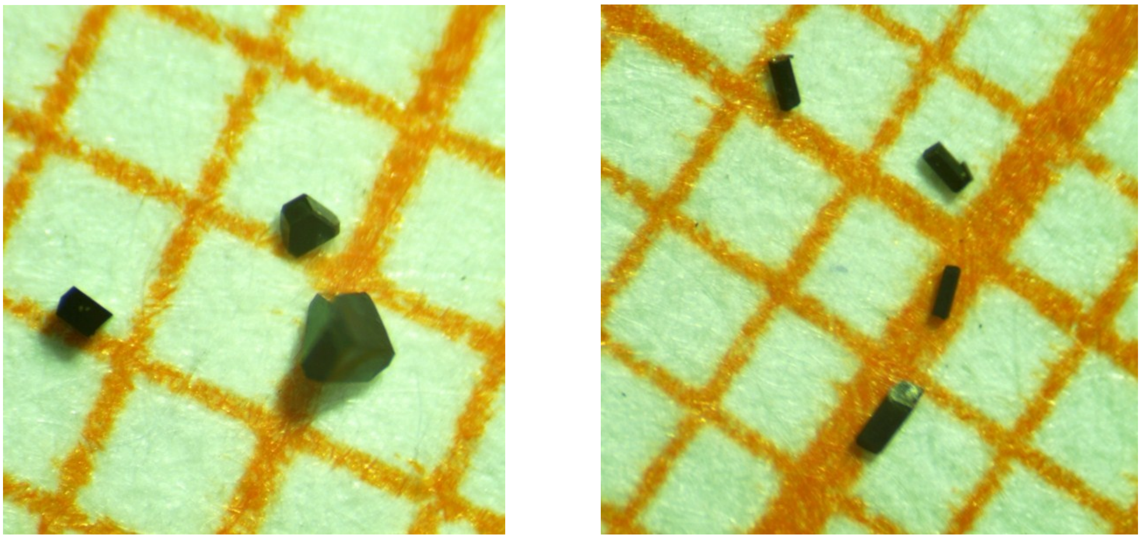

**Fig. S1** The photographs of the CT complexes: left panel for **(TTF1)**•$(I_3)$•$(I_2)$ and right panel for **(TTF2)**•$(I_5)$•$(I_2)$.

**Table S1.** Selected crystallographic data for the CT complexes

| Complexes | **(TTF1)**•($I_3$)•($I_2$) | **(TTF2)**•($I_5$)•($I_2$) |
|---|---|---|
| CCDC number | 1818732 | 1818736 |
| Empirical formula | $C_{30}H_{16}F_4I_5S_8$ | $C_{30}H_{16}F_4I_7S_8$ |
| Formula weight | 1470.31 | 1597.21 |
| Temperature [K] | 173 | 173 |
| $\lambda$ [Å] | 0.71073 | 0.71073 |
| Crystal size [mm$^3$] | 0.3 × 0.2 × 0.1 | 0.2 × 0.1 × 0.1 |
| Crystal system | Triclinic | Monoclinic |
| space group | *P*-1 | *C2/c* |
| *a* [Å] | 12.3537(6) | 24.2826(12) |
| *b* [Å] | 13.4244(10) | 10.2755(8) |
| *c* [Å] | 13.8282(10) | 17.1748(10) |
| $\alpha$ [°] | 61.486(7) | 90 |
| $\beta$ [°] | 76.191(5) | 96.201(5) |
| $\gamma$ [°] | 83.590(5) | 90 |
| *V* [Å$^3$] | 1956.9(3) | 4260.3(5) |
| Z | 2 | 4 |
| $d_{calc}$ [g·cm$^{-3}$] | 3.743 | 2.490 |
| $\mu$ [mm$^{-1}$] | 7.849 | 5.533 |
| $2\theta_{max}$ [°] | 57.188 | 52.044 |
| | $-16 \le h \le 15$ | $-29 \le h \le 29$ |
| Limiting indices | $-16 \le k \le 10$ | $-12 \le k \le 5$ |
| | $-18 \le l \le 14$ | $-21 \le l \le 19$ |
| Data /restraints / parameters | 8811 / 0 / 427 | 4159/30/ 231 |
| *GooF* | 1.026 | 1.181 |
| *R* [$I$>2$\sigma$($I$)] | 0.0517 | 0.0911 |
| $wR_2$ | 0.1060 | 0.1612 |

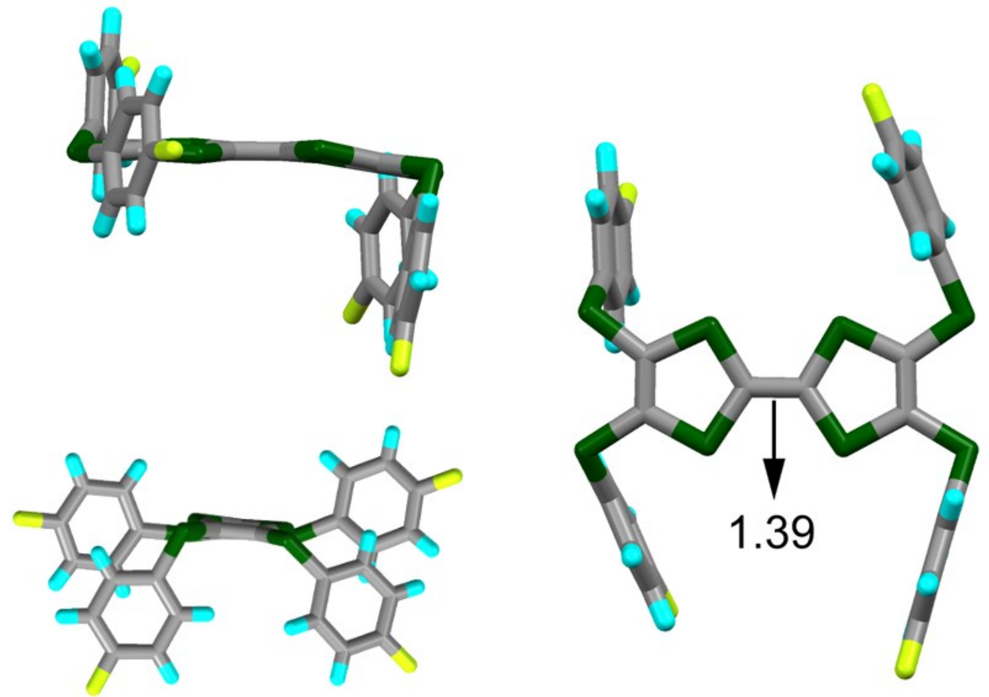


**Fig. S2** The molecular structures of **TTF1** in complex (**TTF1**)•$(I_3)$•$(I_2)$ viewed along the different axes of central TTF core, and the numeric data show the bond length of central C=C bond on the TTF core. The **TTF1** molecule in the complex carry the charge of +1, and the polyiodide is 2-D network formed by $(I_3)^-$ and neutral $I_2$.

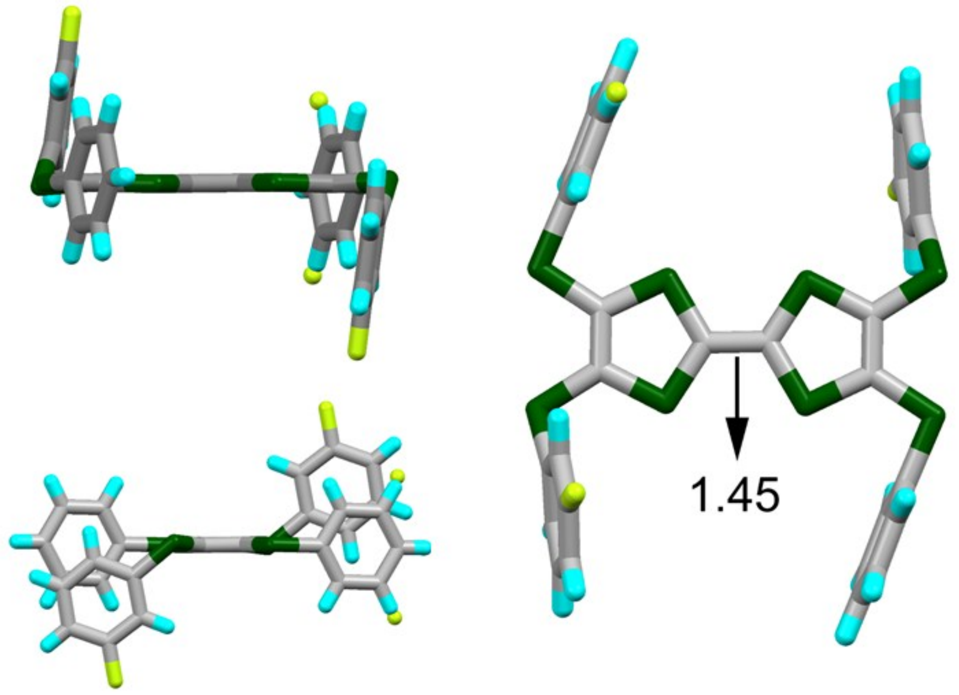


**Fig. S3** The molecular structures of **TTF2** in complex (**TTF2**)•$(I_5)$•$(I_2)$ viewed along the different axes of central TTF core, and the numeric data show the bond length of central C=C bond on the TTF core. The **TTF2** molecule in the complex carry the charge of +2, and the anion is infinite chain of $[(I_n)^-]_\infty$.

**Table S2.** Preparation conditions, stoichiometry formula, habits, yields, and the charges on TTF moieties of the complexes

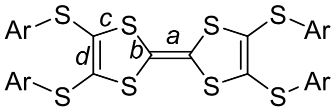

**Table 2.** Preparation conditions, stoichiometry formula, habits, yields, and the charges on TTF moieties of the complexes

| Preparation of the complexes | | | | | | Bond lengths and charge on TTFs | | | | | |
|---|---|---|---|---|---|---|---|---|---|---|---|
| Ar-S-TTFs[a] | $I_2$[a] | Solvents[b] | Composition[c] | Habit[d] | Yield | *a* [Å] | *b* [Å] | *c* [Å] | *d* [Å] | *δ* | *ρ*[e] |
| **TTF1**, 7.1 mg | 15 mg | $CH_2Cl_2$/*n*-hexane | (**TTF1**)•($I_3$)•($I_2$) | block | 3 mg, 22% | 1.392 | 1.721 | 1.741 | 1.350 | 0.721 | +0.97[+1][f] |
| **TTF2**, 7.1 mg | 15 mg | $CH_2Cl_2$/*n*-hexane | (**TTF2**)•($I_5$)•($I_2$) | block | 8 mg, 50% | 1.445 | 1.691 | 1.699 | 1.372 | 0.573 | +2.07[+2][f] |

[a] Ar-S-TTFs ($10^{-5}$ mol) and $I_2$ ($6\times10^{-5}$ mol). [b] $CH_2Cl_2$ (12 mL) and *n*-hexane (3 mL). [c] The composition was determined on the basis of X-ray single crystal structure analyses. [d] The complexes are all in the black colour. [e] The site charge ($\rho$) on TTF moiety could be estimated through an empirical formula $\rho = 6.347 - 7.436\delta$, where $\delta = (b+c) - (a+d)$, and *a*, *b*, *c*, and *d* are bond lengths on TTF moiety.[f] The numeric data in the square brackets are the integer charges on TTF moieties.

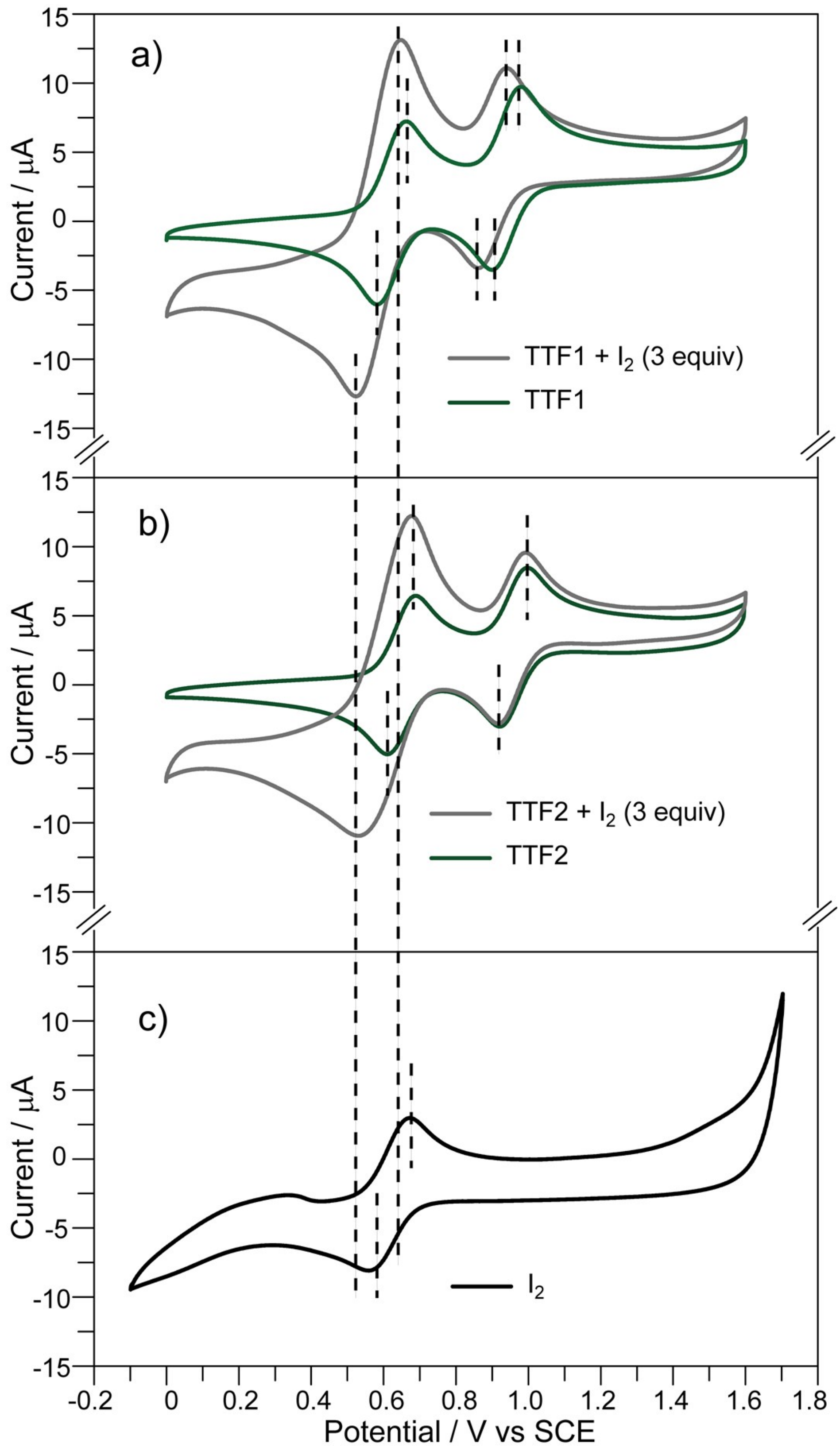


**Fig. S4** Cyclic voltammograms of a) **TTF1** in the presence and absence of $I_2$, b) **TTF2** in the presence and absence of $I_2$, and c) $I_2$. The dashed lines are guides of eye to recognize the oxidation and reduction peaks.
